# A Multimodal Generative AI Framework for Predicting the Toxicity of Nanoparticles

**DOI:** 10.3390/nano16150912

**Published:** 2026-07-24

**Authors:** Leonid Legashev, Arthur Zhigalov, Irina Bolodurina, Alexander Shukhman, Ivan Khokhlov, Svetlana Kolesnik

**Affiliations:** Research Institute of Digital Intelligent Technologies, Orenburg State University Named After V.A. Bondarenko, Pobedy Pr. 13, Orenburg 460018, Russia; leroy137.artur@gmail.com (A.Z.); ipbolodurina@yandex.ru (I.B.); shukhman@gmail.com (A.S.); iv.hohlov-01@yandex.ru (I.K.); svkolesnik_osu@mail.ru (S.K.)

**Keywords:** generative AI, diffusion models, nanotoxicity, molecular dynamics, silver nanoparticles, statistical distribution

## Abstract

Predicting the cytotoxicity of engineered nanoparticles remains a significant challenge due to the vast combinatorial diversity of their physicochemical properties. In this study, we developed a multimodal generative framework to synthesize high-fidelity nanoparticle candidates with predefined toxicity indices. We used a large language model to extract heterogeneous data from scientific articles and utilized SciBERT-based embeddings to encode unstructured textual toxicity summaries. Four generative architectures—CTGAN, TVAE, WGAN-GP, and TabDDPM—were benchmarked using the Synthetic Data Vault quality score. The TabDDPM demonstrated superior performance in capturing complex structure–activity relationships, achieving an SDV quality score of 0.78. The case study validation and feature evolution analysis prove the practical efficacy of the TabDDPM. To validate the physical plausibility of the best generated model, we conducted coarse-grained molecular dynamics simulations in the GROMACS 2026.0 engine using the Martini 3.0.0 force field. Comparative analysis of safe and toxic nanoparticles candidates revealed that the toxic variant induced 2.4 times higher electrostatic stress (88.76 kJ/mol) and significantly prolonged membrane equilibration times. The safe candidates had a lower center-of-mass distance between the nanoparticle and the hydrophobic core of the lipid bilayer compared to the toxic counterpart. These results confirm that the proposed generative approach not only replicates statistical distributions but also captures the underlying biophysical mechanisms of membrane disruption, providing a potentially robust tool for the in silico design of biocompatible nanomaterials.

## 1. Introduction

The rapid integration of nanotechnology into consumer products, medicine, and industrial processes has led to an unprecedented proliferation of engineered nanoparticles (NPs) [1]. Despite their transformative potential, the inherent physicochemical complexity of nanomaterials presents a significant challenge for traditional toxicological assessment [2]. Small variations in core composition, surface functionalization, size, and geometry can lead to radically different biological outcomes, ranging from complete biocompatibility to severe cellular disruption. Conventional in vitro and in vivo screening methods, while accurate, are inherently resource-intensive and low-throughput, creating a critical bottleneck in the “safety-by-design” development of next-generation nanomaterials [3]. In recent years, computational toxicology and in silico modeling have emerged as vital tools for predicting the biological impact of NPs. However, traditional Quantitative Structure–Activity Relationship (QSAR) models often struggle with the high-dimensional and heterogeneous nature of nanotoxicity data, which frequently includes unstructured textual descriptions and complex non-linear correlations. While generative artificial intelligence (AI) has shown promise in drug discovery, its application in the synthesis of physically plausible, toxicologically-tailored nanoparticle specifications remains underexplored [4]. There is an urgent need for robust frameworks that can bridge the gap between large-scale data extraction and high-fidelity biophysical validation. Consequently, the field is shifting toward Quantitative Nanostructure–Activity Relationship (QNAR) models and advanced machine learning (ML) architectures to facilitate high-throughput toxicity screening [5]. These predictive tools leverage critical physicochemical descriptors—including hydrodynamic diameter, surface zeta potential, and core composition—to predict biological endpoints such as oxidative stress, membrane disruption, and pro-inflammatory responses.

The rest of this article is organized as follows. Section 2 provides a literature review on the assessment of nanoparticle toxicity and the use of generative AI models in nano-biology. Section 3 describes the materials and methods used to analyze gathered experimental data and train state-of-the-art generative models. Section 4 provides comparison results, a case study, feature evolution analysis and in silico validity proof of the TabDDPM’s ability to capture complex structure–activity relationships. Section 5 presents a discussion of the results. Section 6 summarizes the conclusions of this study, while Section 7 outlines the corresponding research limitations.

## 2. Literature Review

The recent literature highlights a diverse array of computational strategies for toxicity assessment. For instance, Yousaf I. [6] investigated specific physicochemical properties as foundational descriptors for AI-driven forecasting across industrial sectors. To mitigate the experimental burden of safety assessments, Rouse et al. [7] explored refined computational characterization methods, while Khakpour et al. [8] employed a multi-view learning approach to model the distribution and bioaccumulation of nanoparticles—a prerequisite for understanding long-term chronic toxicity. Furthermore, automated systems utilizing self-supervised learning have been proposed by Lautenschlager et al. [9] to provide rapid, cost-effective evaluations of novel nanomaterials.

A significant focus of contemporary research involves the “protein corona”—the biological interface that dictates a nanoparticle’s toxicological fate. Pipelines have been developed to predict corona composition within living tissues [10], emphasizing how this interface determines eventual bioactivity and toxicity in physiological fluids [11]. Advancements in this area include the introduction of massive datasets and foundation models designed to predict complex nanomaterial–protein interactions [12]. To enhance the precision of these models, researchers have introduced theoretical descriptors based on electronic structure and reactivity [13], as well as multi-task learning frameworks that simultaneously predict multiple toxicity endpoints while offering explainable insights into driving molecular features [14].

Non-linear mapping techniques, such as Bayesian multiple regression trees, have been implemented to track how size and surface charge influence cell viability over time [15]. Additionally, the utility of generative AI is being explored for “safe-by-design” synthesis, allowing for toxicity prediction prior to material fabrication [16]. This shift toward model interpretability aims to move beyond simple prediction to reveal underlying physical mechanisms, such as band gap and surface energy [17].

Data representation and architecture selection remain critical challenges. Studies have evaluated various encoding methods to ensure complex structural nuances are captured for ML input [18], including the use of topology-based descriptors for 3D structural complexity [19]. Comparative benchmarks have further demonstrated the superiority of Convolutional Neural Networks (CNNs) over traditional ML methods in handling high-dimensional toxicological data [20]. To address the prevalence of incomplete datasets, researchers have utilized ML to infer toxicity from partial physicochemical profiles [21] and established links between synthesis parameters and final morphology to enable early-stage toxicity forecasting [22]. Furthermore, the impact of environmental factors, such as temperature, on structural integrity and biological activity has been documented [23], alongside the use of synthetic data to enhance the resolution of nanoparticle identification in complex biological imaging [24].

Recent innovations continue to push the boundaries of predictive accuracy through multimodal architectures that integrate chemical structures with biological assay data [25]. Scalable alternatives to traditional screening now include chemical language models (SMILES-based) [26,27] and AI tools capable of predicting protein ensembles critical to corona formation [28]. To ensure physical plausibility, researchers are combining graph neural networks with physics-based constraints [29], while “parameter-free” ML approaches (e.g., POEM) have been introduced to simplify model selection without sacrificing predictive power [30]. Finally, the integration of multiscale simulations—from atomic to mesoscale—allows for the prediction of collective nanoparticle behavior in solution [31]. Advanced characterization techniques now include modeling the structural evolution of particles [32], enhancing automated microscopy analysis [33], and shifting focus toward transcriptomic-level gene expression profiles to detect toxic responses at the molecular level [34].

Significant research efforts have been directed toward the generation of nanoparticles with tailored properties using advanced generative AI architectures. For instance, Mihandoost et al. [35] explored generative adversarial network (GAN) approaches to predict nanoparticle size within microfluidic systems, while Zi et al. [36] evaluated the performance of cGAN, cWGAN, and WGAN-GP frameworks for predicting the size of silver nanoparticles. Furthermore, Ajith et al. [37] introduced an advanced F-ANcGAN architecture designed to synthesize high-fidelity glioblastoma cell images of nanoparticles. Beyond morphological synthesis, Wang et al. [38] utilized generative machine learning to model the evolution of grain boundary networks in nanocrystalline materials under mechanical stress. More recently, Benaatou et al. [39] developed a constraint-aware conditional generative framework for the computational inverse design of biofunctional polymer coatings. To the best of our knowledge, there is currently a lack of research specifically focused on the generation of nanoparticles with a targeted toxicity index. We believe our study addresses this gap, providing a novel contribution to this rapidly evolving field. Table 1 summarizes the key findings and architectures identified in previous studies regarding the application of generative AI in nanoparticle synthesis.

The scientific novelty of this research lies in the development of a multimodal generative pipeline that integrates Large Language Models (LLMs) for automated data extraction with advanced Denoising Diffusion Probabilistic Models (TabDDPMs) for material synthesis. Unlike previous models that rely solely on numerical descriptors, our approach incorporates high-dimensional textual embeddings to capture the nuanced toxicological summaries found in the scientific literature. Furthermore, we implement a novel inverse-scaling and decoding framework that allows for the precise recovery of categorical and physical properties from a compressed latent space, ensuring that generated candidates are not only statistically accurate but physically interpretable.

The significance of this work stems from the successful cross-scale validation of AI-generated predictions using Molecular Dynamics (MD) simulations. By demonstrating that candidates generated at the extremes of the toxicity spectrum (toxicity index of 0.1 vs. 0.9) exhibit quantifiable differences in membrane interaction energies and system stability within the GROMACS (version 2026.0, Science for Life Laboratory, Stockholm, Sweden) engine environment [40], we provide a physical foundation for synthetic data. This provides a methodology for the rapid in silico prototyping of nanoparticles, potentially reducing reliance on animal testing and accelerating the identification of safe-by-design parameters for biomedical applications. We also implemented a nanoparticle visual passport system to generate parametric morphological projections that mimic the output of electron microscopy to qualitatively assess the physical plausibility of the generated candidates as a “human-in-the-loop” validation step.

Our main contributions to the field are three-fold:

1. Methodological: We propose a scalable architecture for extracting and normalizing heterogeneous nanotoxicological data using a state-of-the-art LLM.
2. Algorithmic: We perform comparative benchmarking of four generative architectures, identifying TabDDPM as the superior model for maintaining correlation fidelity in small-scale, complex nanoparticle datasets.
3. Experimental: We integrate diffusion-based tabular generation with Martini 3.0.0 coarse-grained MD simulations and perform case study validation, establishing a link between generative AI outputs and correct biophysical behavior.

## 3. Materials and Methods

**Figure 1:**
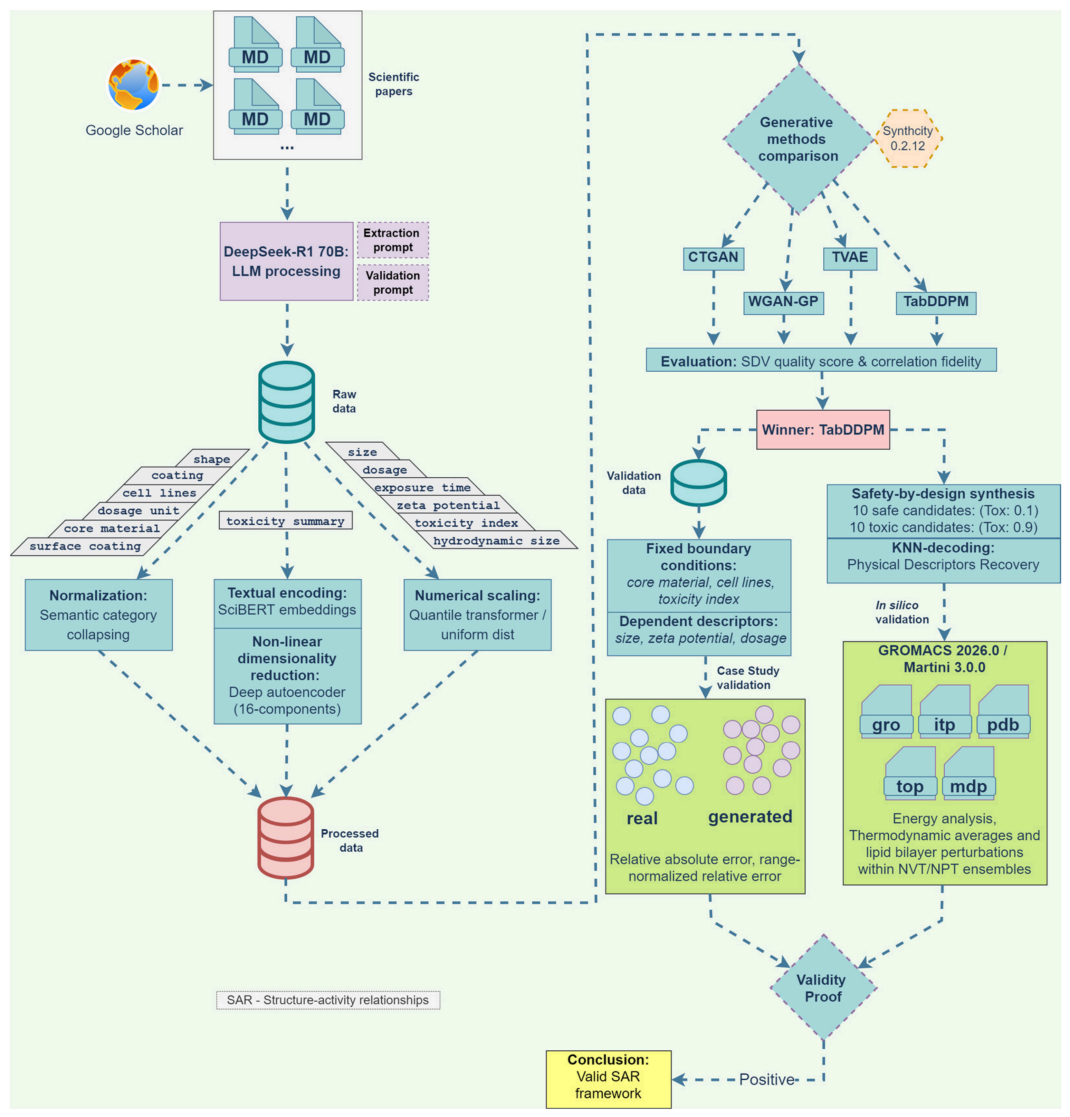
Research pipeline of AI-driven generative nanotoxicology.

The general research pipeline of the proposed AI-driven generative nanotoxicology approach is presented in Figure 1.

### 3.1. Data Gathering and Preprocessing

We curated a comprehensive dataset by extracting information from over 250 peer-reviewed publications concerning nanoparticle toxicity. To facilitate machine readability, unstructured text was converted into Markdown (.md) format. We employed a large language model, Deepseek-r1 70B, hosted on a private server, utilizing a dual-prompt strategy:

1. An extraction prompt designed to capture specific physicochemical parameters (e.g., size, shape, zeta potential), generate a qualitative toxicity summary, and assign a normalized toxicity index ($I_{tox}\in [0,1]$) based on toxicity summary. The extraction prompt is listed in Appendix A.1.

2. A validation prompt to ensure the fidelity and consistency of the extracted data against the source material. The validation prompt is listed in Appendix A.2.

**Figure 2:**
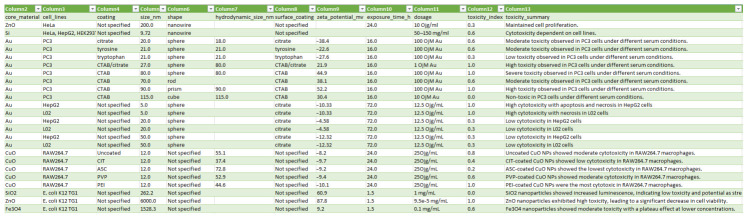
A fragment of raw data captured by the Deepseek-r1 70B model.

This process yielded a structured dataset of 1225 unique records. A fragment of the raw gathered data is shown in Figure 2. Due to the scale of the obtained dataset, manual verification of every record was prohibitive. To ensure the reliability of the LLM-based extraction, we conducted iterative pilot validation during the prompt-tuning phase using a control subset of 50 randomly selected publications. Notably, this subset included a benchmark against our own experimental study published in *Toxics* [41], which investigated the toxicity profiles of three distinct nanoparticle types: ZnO, Fe_3_O_4_ and SiO_2_. The Deepseek-r1 70B model successfully extracted and cataloged the parameters for all three nanoparticles with high fidelity, demonstrating the robustness of the proposed automated pipeline. It should be noted that the raw data contains gaps and minor inaccuracies in some records.

Let $D={\left\{(x_{i},y_{i})\right\}}_{i=1}^{N},$ where N=1225 is the number of samples. Each sample consists of a feature vector x and a target variable *y* (the toxicity index). The feature vector is decomposed into

(1) $$x=[x_{num},x_{cat},x_{text}],$$

where $x_{num}\in {\mathbb{Z}}^{d}$ are continuous physicochemical properties (size, dosage value, zeta potential, hydrodynamic size, exposure time); $x_{cat}\in l$ are categorical descriptors (core material, cell lines, shape, coating, surface coating and dosage unit); $x_{text}\in {\mathbb{R}}^{16}$ is the reduced latent representation of the toxicity summary.

Initial data cleaning involved the removal of non-informative metadata (e.g., DOI, verification status). The dosage feature was split into dosage unit and dosage value columns. The following partial standardization was enforced: equivalent mass-volume metrics, including μg/mL, ug/mL, mg/L and ppm, were algorithmically unified into a single baseline unit, ug_ml. Other non-reducible dimensions like mM, nM and mg/kg were preserved as individual categories, ensuring the machine learning architecture evaluates numerical values strictly within the boundaries of their respective physical dimensions. To ensure chemical consistency, categorical features $x_{cat}$ were subjected to semantic normalization and rare-category collapsing to improve model convergence. First, unstructured textual labels were mapped to canonical classes using deterministic keyword-matching dictionaries to unify synonyms, chemical formulas, and typographical variants. Second, statistical regularization was applied to the consolidated categorical distributions: any rare sub-category with an absolute frequency threshold of *N* < 5 was automatically collapsed into an aggregated other category. The resulting collapsed categories are presented in Table 2.

To address the heavy-tailed distributions and physical outliers (e.g., hydrodynamic size and exposure time), we employed a quantile transformation strategy. Numerical features were mapped to a uniform distribution (output_distribution = ’uniform’) using a robust quantile-based scaler, ${\hat{x}}_{num}=F_{quantile}(x_{num}),$ where *F* maps the empirical distribution to U(0,1). Missing values in numerical attributes were handled via median imputation, which preserves the integrity of the data distribution better than mean-based methods in the presence of extreme physicochemical values.

For complex, unstructured descriptions, we utilized a Bio-Medical Language Model approach. Textual feature toxicity summary was transformed into 768-dimensional vectors using the SciBERT (Allen Institute for AI, Seattle, WA, USA) architecture (https://github.com/allenai/scibert, accessed on 10 February 2026), which was pre-trained on a large corpus of scientific literature. To facilitate efficient learning within the generative frameworks, these high-dimensional embeddings were subsequently compressed into a dense, low-dimensional manifold using a parametric deep non-linear Autoencoder architecture. The compression model consists of an encoder network *f_ϕ_* and a decoder network *g_θ_*, both utilizing parametric fully connected layers interleaved with non-linear Rectified Linear Unit (ReLU) activation functions. The encoder progressively compresses the original input embedding 768-dimensional vector through a hidden projection layer down to a 16-dimensional bottleneck latent representation. Optimization was performed over 50 training epochs with a batch size of 32 using the Adam optimizer at a static learning rate of 1 × 10^−3^. The objective function minimized the Mean Squared Error (MSE) loss between the empirical inputs and their subsequent reconstructions.

### 3.2. Generative Models

Four state-of-the-art generative architectures were evaluated for their ability to synthesize physically plausible nanoparticles:

1. Conditional Tabular GAN (CTGAN) [42]: This architecture employs a conditional generator and mode-specific normalization to effectively handle imbalanced categorical features and multimodal continuous distributions common in toxicological datasets.
2. Tabular Variational Autoencoder (TVAE) [42]: An adaptation of the variational autoencoder framework for tabular data, TVAE utilizes a specialized encoder–decoder structure to map nanoparticle properties into a latent space, optimizing the evidence lower bound to capture complex joint distributions.
3. Wasserstein GAN with Gradient Penalty (WGAN-GP) [43]: This model integrates the Wasserstein distance with a gradient penalty term to stabilize the adversarial training process and mitigate mode collapse, ensuring a more diverse and physically realistic synthesis of nanoparticle parameters.
4. Tabular Denoising Diffusion Probabilistic Model (TabDDPM) [44]: A state-of-the-art diffusion-based architecture that generates synthetic samples by iteratively reversing a Gaussian noise process for numerical features and a multinomial process for categorical descriptors, providing superior fidelity in modeling intricate data dependencies.

The goal of the generative models is to learn a parameterized distribution $P_{\theta }(x,y)$ that approximates the true underlying distribution $P_{data}(x,y)$. For the TabDDPM, the process is defined by a forward Gaussian transition *q*:

(2) $$q(x_{t}|x_{t-1})=N(x_{t};\sqrt{1-\beta_{t}}x_{t-1},\beta_{t}I),,$$

where t∈{1,2,…,T} is an integer scalar index, *T* is the total length of the diffusion chain, I is a standard identity matrix, $\beta_{t}$ is the variance coordinate schedule at step *t*; and a learned reverse denoising process:

(3) $$p_{\theta }(x_{t-1}|x_{t})=N(x_{t-1};\mu_{\theta }(x_{t},t),\sum_{\theta }\left(x_{t},t\right)).$$

Selected generative models were benchmarked using the Synthetic Data Vault (SDV) quality score *Q* of SDV library (version 1.15.0, DataCebo Inc., Boston, MA, USA), which is an aggregate of the Marginal Distribution Fit ($S_{dist}$) and Correlation Similarity ($S_{corr}$):

(4) $$Q=\frac{S_{dist}+S_{corr}}{2},$$

where Q∈[0,1]. The Marginal Distribution Fit (shape similarity) $S_{dist}$ score is the average of all single-column scores:

(5) $$S_{dist}=\frac{1}{x_{num}+x_{cat}}\left(\sum_{i=1}^{x_{num}}{\mathrm{Score}}_{\mathrm{KS}}(i)+\sum_{j=1}^{x_{cat}}{\mathrm{Score}}_{\chi^{2}}(j)\right),$$

where ${\mathrm{Score}}_{\mathrm{KS}}$ is the Kolmogorov–Smirnov complementary score, and ${\mathrm{Score}}_{\chi^{2}}$ is the Chi-Squared complementary score.

The Correlation Similarity (pairwise correlation) $S_{corr}$ score measures the fidelity of joint probabilities and bivariate dependencies across all possible unique column pairs:

(6) $$S_{corr}=\frac{1}{N_{pairs}}\sum_{m<n}\left(1-\left|R_{real}(m,n)-R_{syn}(m,n)\right|\right),$$

where $R_{real}$ and $R_{syn}$ are correlation matrices.

To optimize the training duration for each generative architecture (CTGAN, TVAE, WGAN-GP and DDPM) and mitigate the risk of overfitting or mode collapse, a validation-driven early stopping algorithm was deployed. The optimal iteration threshold was established by evaluating the statistical utility of the synthetic outputs at incremental steps of 100 training epochs. At each step, a synthetic dataset was generated and benchmarked against the empirical data using the SDV score. The algorithm monitored the validation score across cycles; if no structural improvement exceeding a significance threshold of ΔScore = 0.001 was recorded for 5 consecutive cycles, the training loop was terminated. The optimal model checkpoint capturing the peak global validation score was then isolated for downstream validation, ensuring robust generalization without over-parameterization. We selected the following optimal iterations values: “ctgan”: *n_iter_* = 500, “tvae”: *n_iter_* = 300, “wgan-gp”: *n_iter_* = 500, “ddpm”: *n_iter_* = 10,000. The train/test split ratio used for this study was 80–20.

The validation of the best-performing generative model’s ability to capture complex structure–activity relationships (SAR) was performed in three ways: a case study validation by pairwise comparison of real and generated nanoparticles; feature evolution analysis of large volumes of generated data, and in silico validation of safe and toxic silver nanoparticles using the GROMACS 2026.0 engine. The generative artificial intelligence Gemini model was used in this paper to assist in the study design of an in silico experiment regarding the preparation of configuration files and conditions for conducting the experiment.

## 4. Experimental Results

### 4.1. Comparison of Generative Models for Inverse Design of Nanoparticles at Specific Biological Toxicity Thresholds

**Figure 3:**
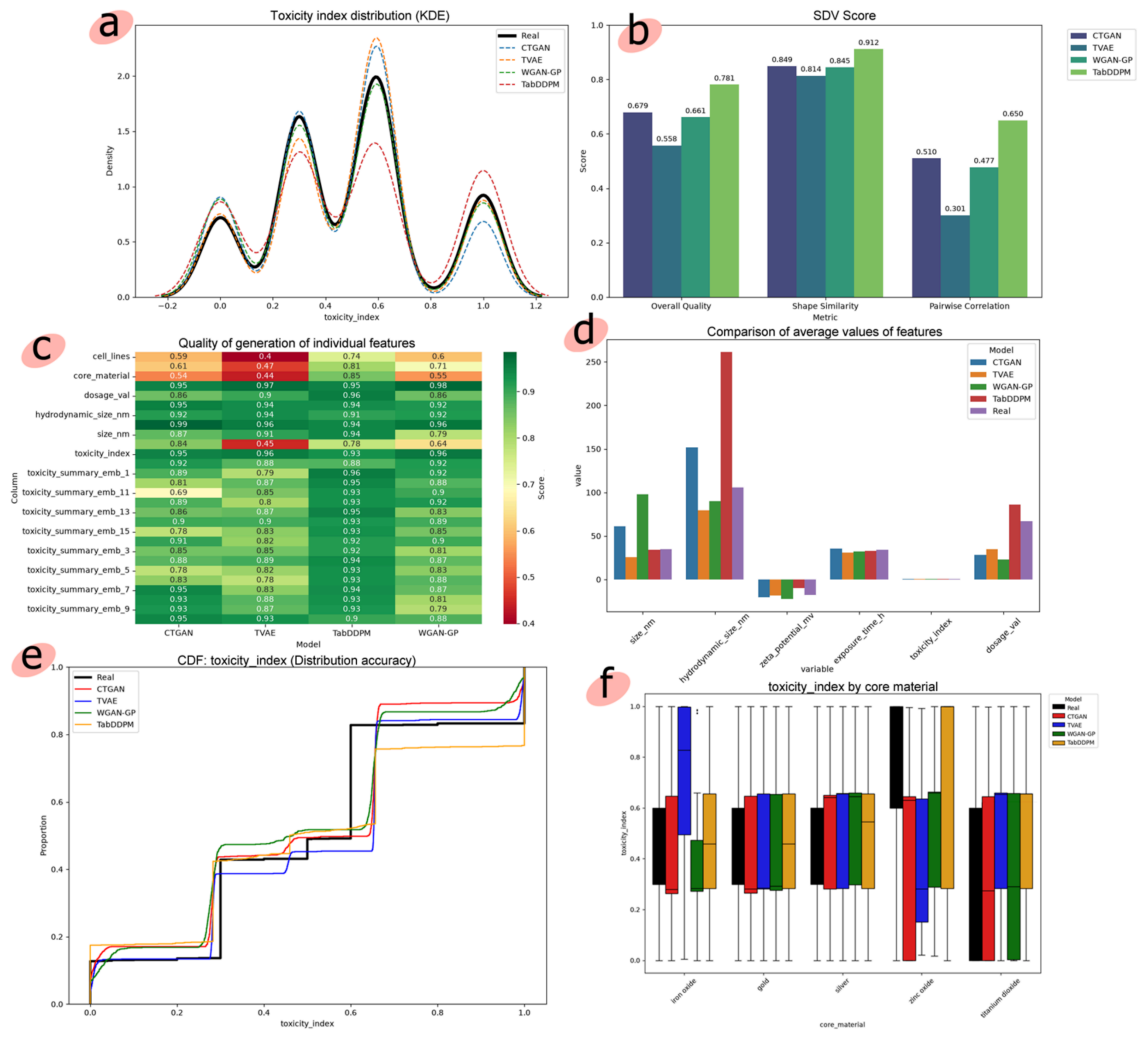
(**a**) KDE distribution of toxicity index, (**b**) SDV score, (**c**) heatmap plot of quality of generation of individual features, (**d**) bar plot of mean values of numerical features, (**e**) empirical cumulative distribution function plot of toxicity index and (**f**) boxplot of toxicity index of five most frequent core materials across four generative models.

**Figure 4:**
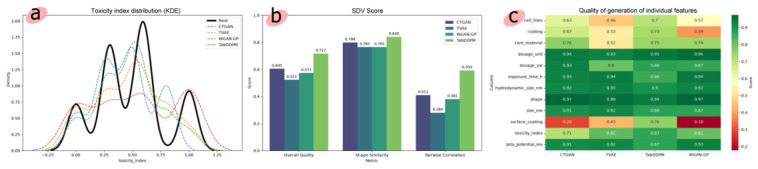
(**a**) KDE distribution of toxicity index, (**b**) SDV score and (**c**) heatmap plot of quality of generation of individual features across four generative models without textual embeddings.

The evaluation of fidelity and stability of four generative models to predict nanoparticle toxicity index based on multimodal data is depicted in Figure 3. The evaluation of fidelity and stability of four generative models to predict nanoparticle toxicity index based on tabular data without toxicity summary textual embeddings is depicted in Figure 4.

In Figure 3a, the Kernel Density Estimation (KDE) of the toxicity index reveals that all four architectures successfully captured the multimodal nature of the empirical data, identifying key toxicity clusters at approximately 0.0, 0.35, 0.6 and 1.0, which correspond to the instructions in the extraction prompt from Appendix A.1. While all models demonstrated high fidelity, TabDDPM exhibited the highest degree of overlap with the ground truth (real data), suggesting superior capability in modeling the underlying probability density function without over-smoothing. In Figure 3b, the overall quality score *Q* identifies TabDDPM as the top-performing model, achieving a value (4) of 0.78. This result is primarily driven by its Column Shape Similarity metric, where it outperformed the adversarial GAN-based and variational VAE-based approaches. CTGAN followed as the second-best performer, while TVAE and WGAN-GP demonstrated lower overall scores, particularly struggling with complex categorical boundaries. In Figure 3c, granular analysis via the quality heatmap confirms that TabDDPM provides the most consistent generation across all feature types, evidenced by a predominantly “green” high-score profile. In contrast, the other three methods exhibited significant performance degradation when handling categorical features, such as surface coating, cell lines, coating, and core material. This indicates that the diffusion-based approach is more robust at maintaining the integrity of discrete semantic categories compared to traditional latent-space models. In Figure 3d, statistical analysis of the mean values reveals a “high-side” bias in the TabDDPM and CTGAN results. Specifically, generated values for hydrodynamic size were consistently higher than the empirical averages. This phenomenon is likely an artifact of the quantile transformation and subsequent clipping logic, where the models attempt to fill the “tails” of the distribution results in a higher frequency of near-maximum physical values compared to the other models. In Figure 3e, all models accurately convey the entire range of toxicity indices without missing extreme values. In Figure 3f, we observe how the models convey toxicity for the five most common core materials in the dataset. The TabDDPM shows better results regarding the capture of toxicity thresholds for zinc oxide core material compared to other models. Quantitative analysis of Figure 3a–c and Figure 4a–c demonstrates that supplementing the TabDDPM pipeline with toxicity summary embeddings systematically improves all structural fidelity benchmarks. Specifically, pairwise correlations rose by 6% (from 0.59 to 0.65), feature shape similarity increased by 7% (from 0.84 to 0.91), and the overall SDV score advanced by 7% (from 0.71 to 0.78).

To quantitatively evaluate the fidelity of the synthetic data generated by the four candidate models (CTGAN, TVAE, WGAN-GP, and TabDDPM), we implemented cluster analysis within the original high-dimensional feature space. We performed a K-Means clustering analysis directly on the scaled empirical feature space comprising 1225 records. The mathematically optimal number of clusters K = 7 was determined objectively by maximizing the global silhouette coefficient across a range of K in [2, 19]. To measure how closely the generated synthetic instances mirror actual empirical entries, we computed the Mean Distance to Real Anchors (MDRA) using the full-dimensional scaled feature vectors via

(7) $$\mathrm{MDRA}=\frac{1}{N_{syn}}\sum_{i=1}^{N_{sym}}\underset{j}{\min}{\left\|x_{syn,i}-x_{real,j}\right\|}_{2}$$

To quantitatively assess the models’ vulnerability to mode collapse or sub-distribution omission (the long-tail data problem), we calculated the sub-distribution mode coverage. A cluster was defined as successfully captured if the minimum Euclidean distance from any synthetic point to that specific real cluster center fell within a normalized a dynamic tolerance radius. To guarantee that the models learn the complex, non-linear cross-correlations, we evaluated the global covariance matrices. We extracted the empirical covariance tensor $\sum_{real}$ and compared it to each synthetic covariance tensor $\sum_{syn}$ across all vectorized features. The structural alignment was quantified using the Covariance Tensor Structural Mismatch Loss, defined as the Frobenius norm of their difference:

(8) $$L_{cov}=\sqrt{\sum_{m}\sum_{n}{\left(\sigma_{real,mn}-\sigma_{syn,mn}\right)}^{2}}$$

**Figure 5:**
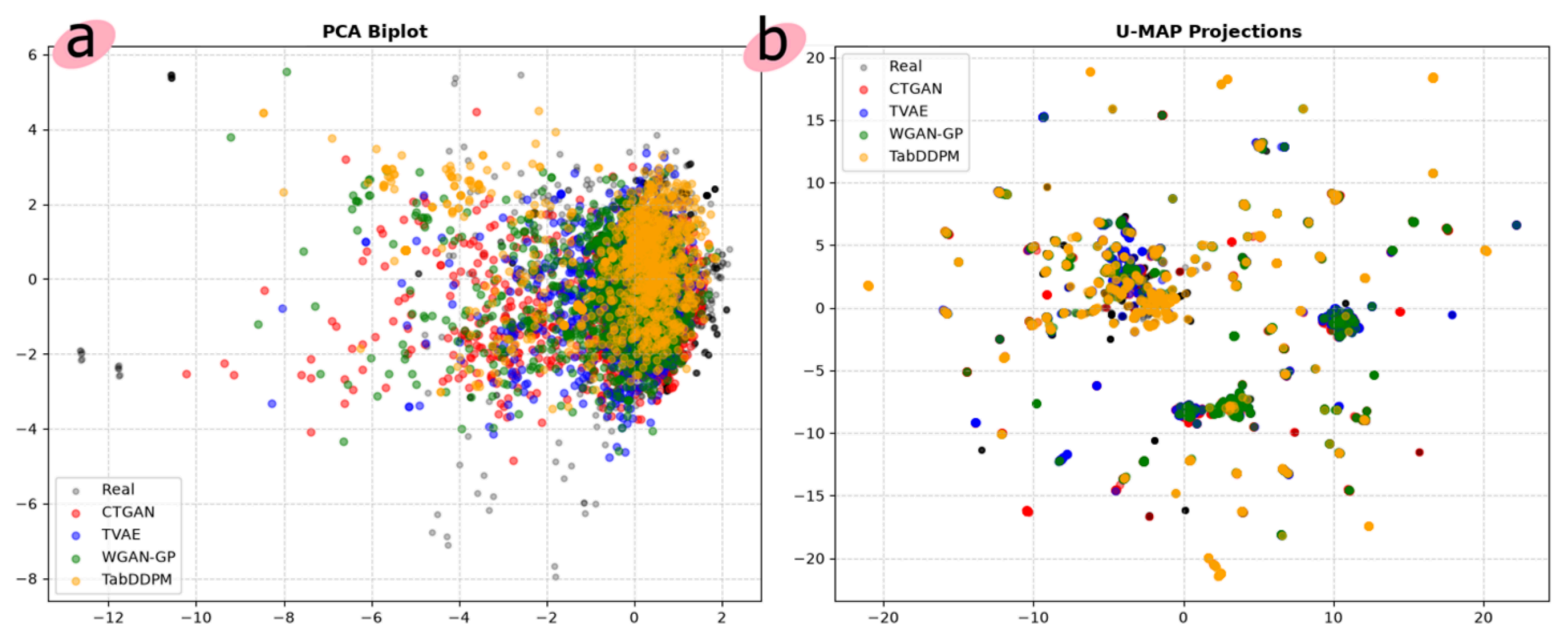
(**a**) PCA and (**b**) U-MAP cluster analysis within the original feature space.

Conducted analysis revealed distinct structural behaviors and capabilities across the four generative frameworks, as presented in Table 3. The visual distribution of the data points is presented in Figure 5.

The unsupervised cluster validation objectively separated the nanotoxicity data into seven granular sub-distributions. For example, Cluster 3 isolated low-toxicity, ultra-small titanium dioxide nanofibers with a size of 10.0 nm and toxicity_index of 0.0, while Cluster 2 captured high-toxicity gold and zinc oxide spherical matrices with a size of 45.3 nm and toxicity_index of 0.78. When evaluating local sample fidelity, TabDDPM outperformed all GAN and VAE variations with an MDRA value of 8.108. However, looking at global manifold coverage, CTGAN successfully captured 71.4% (5 out of 7 clusters) of the distinct sub-distributions, showcasing a strong ability to map wide boundaries, though at the expense of local sample accuracy. TabDDPM achieved the lowest covariance mismatch score of *L_cov_* = 37.643. This indicates that TabDDPM preserves the complex physical and biological relationships with robust fidelity. It should be noted that despite the strong local fidelity and covariance alignment demonstrated by the top-performing generative models, a granular inspection of the high-dimensional manifold from Figure 5a,b reveals localized structural coverage gaps where certain real data points lack synthetic counterparts. These localized gaps are driven by the high combinatorial cardinality of nanomaterial attributes, where unique, rare pairings of specific core compositions, shapes, cells lines and surface coatings occur only a handful of times across the baseline dataset of 1225 records. For these ultra-sparse configurations, the generative models probably lack the critical mass of sample density required to safely interpolate the manifold boundary.

**Figure 6:**
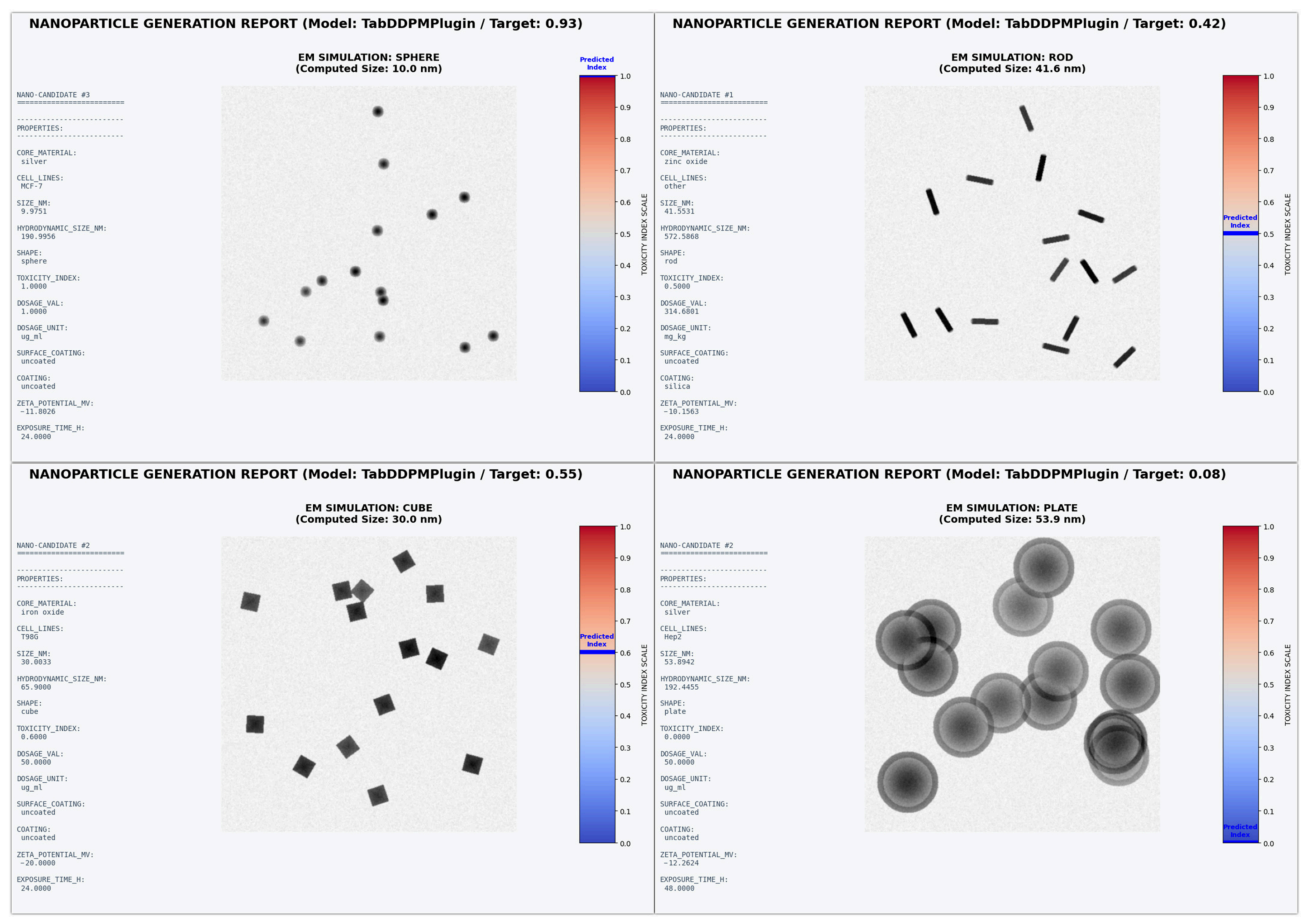
Visualization examples of four generated nanoparticles with parametric morphological projection.

To facilitate the rapid interpretation and visual validation of AI-generated candidates, we implemented a nanoparticle passport system to generate parametric morphological projections that mimic the output of Electron Microscopy (EM). By mapping the generated geometric parameters (e.g., spherical, rod-like, cube-like, etc.) and the calculated diameter onto a grayscale noise-interpolated canvas, the system provides an immediate visual proxy for the nanoparticle’s morphology. Selective visualization of the generated outputs of the TabDDPM is presented in Figure 6. Such a nanoparticle passport allows researchers to qualitatively assess the physical plausibility of the generated dimensions relative to the predicted shape, acting as a “human-in-the-loop” validation step.

### 4.2. Case Study Validation of TabDDPM

To assess the practical reliability of the TabDDPM, we conducted a targeted fidelity analysis by comparing the experimental data of real nanoparticles with generated candidates under fixed boundary conditions (core material, cell lines, and toxicity index), while size, zeta potential and dosage were dependent descriptors. We prepared an additional validation dataset by gathering nanotoxicity data from an additional 20 scientific papers using the Deepseek-r1 70B model. From this dataset, we extracted several records of core material and cell line pairs and obtained their toxicity indices. We generated several thousand nanoparticles using the TabDDPM and selected the best candidate that most closely approximated the dependent descriptors of the real nanoparticle. The comparison results are presented in Table 4. To correctly assess the size and dosage values, we used the standard relative absolute error formula:

(9) $$\mathrm{RE}=\frac{\left|X_{gen}-X_{real}\right|}{\left|X_{real}\right|}$$

Standard relative error is unsuitable for zeta potential evaluation because it can cross zero or flip signs. Therefore, we used the range-normalized relative error by dividing the absolute difference by the total empirical range (Δ*R* = 109.2) of zeta potentials observed across all 12 real nanoparticles:

(10) $${\mathrm{RE}}_{\mathrm{norm}}=\frac{\left|X_{gen}-X_{real}\right|}{\Delta R}$$

The error metrics calculations for case study validation are presented in Table 5.

**Figure 7:**
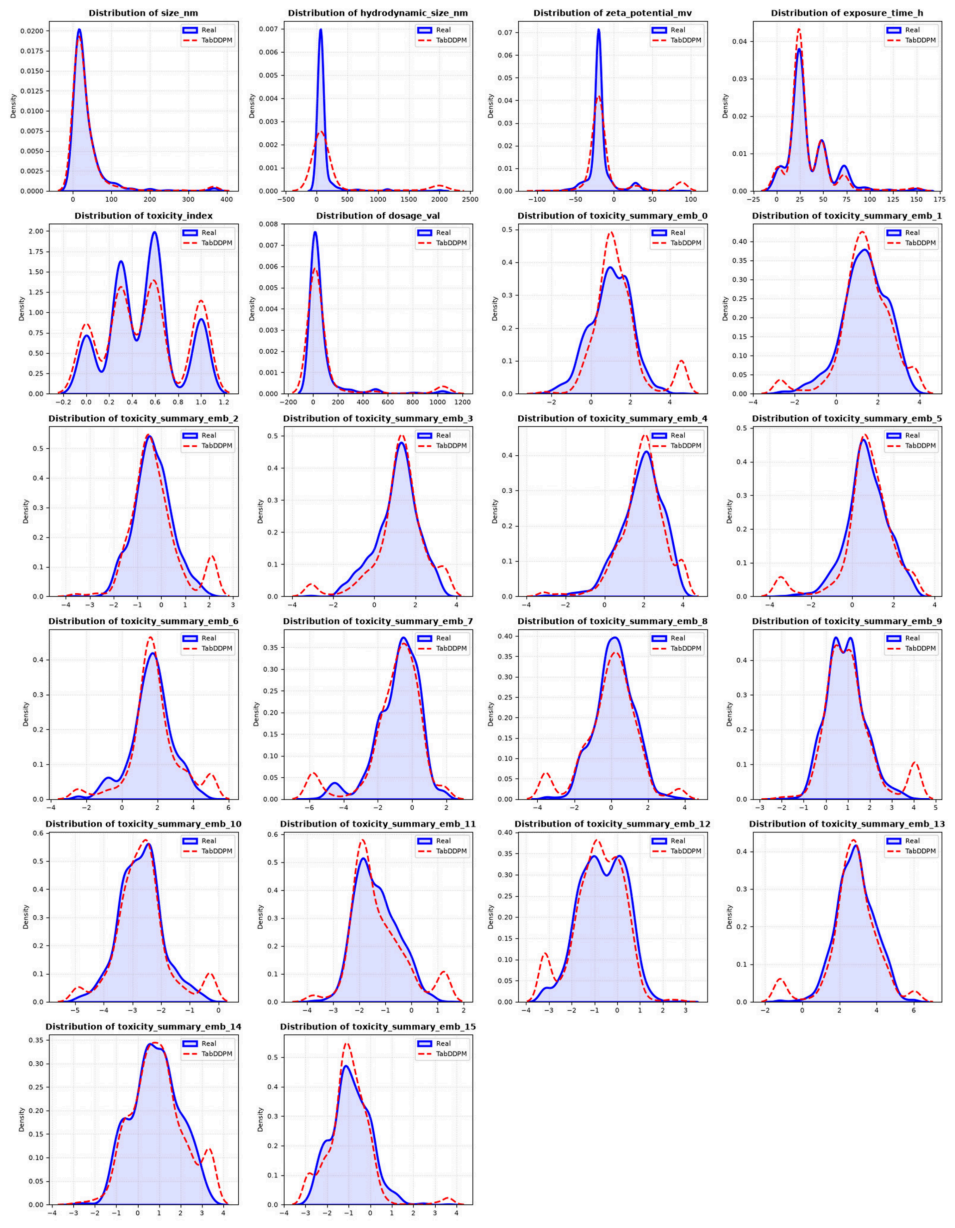
Statistical distribution of real and data generated by the TabDDPM.

The generative framework demonstrated moderately high predictive accuracy across the validation data. While the general trend across the 12 verified external nanoparticles confirmed close alignment, minor structural deviations were observed in specific regions of the feature space: zeta potential deviation may occur because highly charged states (<−40 mV) represent an under-sampled region of the experimental manifold, forcing the diffusion process to extrapolate along a steep mathematical gradient. Variance in size highlights the model’s localized smoothing behavior when operating at the upper boundaries (>300 nm), where the empirical data density is naturally sparse. We can observe from Figure 3d that the average zeta potential of real data is approximately −20.0 mV, representing a characteristic stability threshold for the majority of synthesized nanoparticles. The TabDDPM exhibits a tendency toward regression to the mean when predicting values for specific outliers. Consequently, in cases where experimental nanoparticles possess higher zeta potentials (e.g., −40.0 mV), the generative process occasionally biases these towards the modal value of −20.0 mV, potentially smoothing out the variance in favor of higher overall statistical likelihood. High discrepancies in absolute relative dosage and size error mainly occur for nanoparticle cases (such as cases 6 and 12) in which cell lines belong to the rare ‘other’ category. The statistical distribution of real data and data generated by the TabDDPM is presented in Figure 7. We can conclude that most numerical data and toxicity summary embeddings in the synthetic data have distributions close to the real data counterparts; higher density peaks can be observed mostly for the zeta potential and hydrodynamic size features in the real data compared to generated data.

### 4.3. Feature Evolution Analysis of TabDDPM

**Figure 8:**
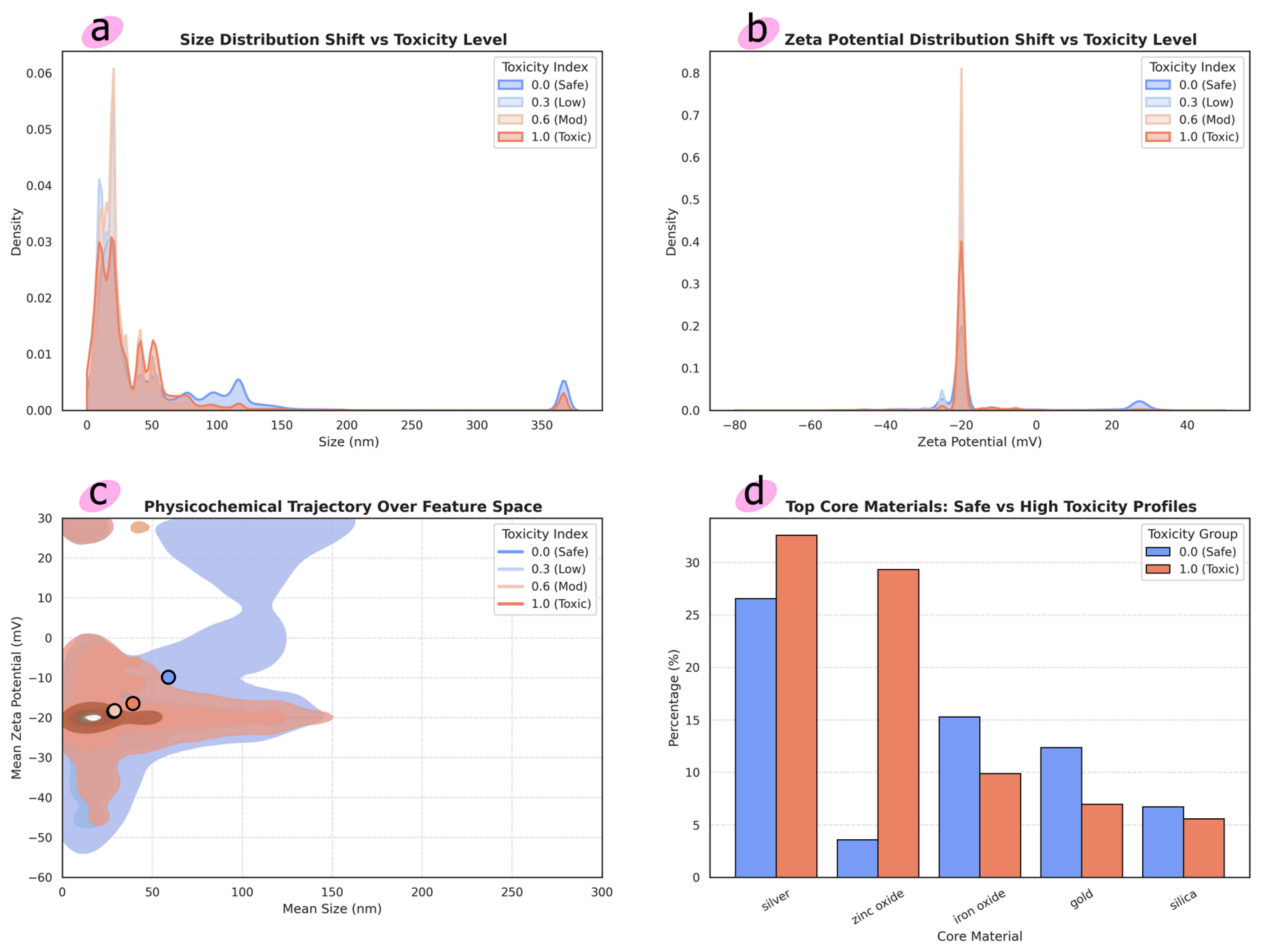
(**a**) KDE ridge plot of size and (**b**) zeta potential; (**c**) joint feature drift plot of size and zeta potential with colored dots indicating the center of each toxicity group; (**d**) bar plot of top core materials in relation to safe and toxic groups.

To evaluate the efficacy of the TabDDPM, we performed feature evolution analysis based on generated data, as shown in Figure 8. Out of the initial 8,000,000 conditionally generated candidate rows (2,000,000 per target toxicity value), a post-generation fidelity filter $\left(\left|I_{tox,gen}-I_{tox,target}\right|<0.05\right)$ isolated a refined dataset of 1,714,711 entries.

To evaluate whether the generative model synthesized distinct physical profiles along the toxicological gradient, we applied the non-parametric Kruskal–Wallis *H*-test [45]. The analysis revealed a statistically significant divergence across the four fixed toxicity levels ($I_{tox}\in \{0.0,0.3,0.6,1.0\}$) for size (*H* = 32,036.91, *p* < 0.001), zeta potential (*H* = 13,849.99, *p* < 0.001) and dosage (*H* = 29,624.81, *p* < 0.001). These test statistics demonstrate that the TabDDPM successfully segmented the underlying data manifold into distinct physical identities mapped directly to toxicity levels.

In Figure 8a, we can observe that the size distribution undergoes a structural shift as toxicity increases. Safe nanoparticles ($I_{tox}=0.0$) exhibit a wider, multimodal size distribution with population extending beyond 100 nm. In Figure 8b, we can observe that for the zeta potential, toxic nanoparticles collapse around a narrow peak centered at approximately −16.4 mV to −18.4 mV, while safe nanoparticles exhibit a broader charge variance and a more neutral mean value. The trajectory analysis in Figure 8c shows that the TabDDPM captures the highly non-linear nature of nano–bio interactions. The overlapping region centered in the [10 nm, 50 nm] range and [−10 mV, −25 mV] range indicates that certain size–charge configurations are shared across all toxicity levels. This overlap demonstrates that size and zeta potential alone do not define a nanoparticle’s toxicity, which can be affected by other unconstrained features, such as surface coating, exposure time, etc. The safe nanoparticle population (light blue region) expands into a broad, asymmetric envelope toward larger core sizes (>60 nm) and neutral-to-positive zeta potentials (>−5 mV). The positions of the mean centroids do not follow a simple linear sequence; instead, they reveal a non-linear parabolic trajectory over the feature space. Moving from the safe to the highly toxic point, the mean size drops from 58.8 nm to 39.2 nm, and the zeta potential drops from −9.8 mV to −16.4 mV. This shift follows the expected trend where decreased size accelerates cellular uptake and enhances cytotoxicity. The centroids for the intermediate states (0.3) and (0.6) overshoot the absolute toxic endpoint, compressing down to smaller average sizes (~28.5 nm) and more negative charges (~−18.4 mV). The reason for this effect may be that inherently less toxic core materials must reach extreme physical states to achieve even moderate toxicity, compensating for their low baseline chemical reactivity. The model’s compositional distribution demonstrates chemical discrimination, as shown in Figure 8d. In the highly toxic cohort ($I_{tox}=1.0$), silver and zinc oxide emerge as the dominant core materials, exceeding their representation in the safe cohort.

### 4.4. In Silico Validation of TabDDPM

To evaluate the predictive accuracy and effectiveness of the TabDDPM, we performed high-resolution molecular simulations using the GROMACS 2026.0 engine. Two distinct sets of high-fidelity candidates (silver core, A549 cell lines and PEG-coated nanoparticles) classified by the TabDDPM as safe (target toxicity index of 0.1) and toxic (target toxicity index of 0.9) were synthesized in silico. The average values of nanoparticle size and zeta potential were calculated. The average zeta potential and size of safe nanoparticles were −23.57 mV and 34.22 nm, respectively. The average zeta potential and size of toxic nanoparticle were −18.03 mV and 18.92 nm, respectively. Generally, a decrease in nanoparticle size correlates with enhanced toxicity, primarily due to the increased surface-area-to-volume ratio, which promotes higher chemical reactivity and facilitates cellular internalization. Highly negative zeta potential values often correlate with increased cytotoxic potential through mechanisms involving electrostatic attraction to membrane components and subsequent induction of oxidative stress. Given the significant physical dimensions of the generated candidates (approximately 20–35 nm in diameter), a Coarse-Grained (CG) approach was employed using the Martini 3.0.0 force field. This allowed for a representative simulation of the interaction between complex nanoparticles and biological membranes at a relevant spatiotemporal scale.

The selection of the specific nanoparticle configuration is based on several critical toxicological and physicochemical considerations:

1. Core material: Silver nanoparticles are among the most studied nanomaterials due to their potent antimicrobial properties and widespread use in consumer products. They serve as a gold standard in nanotoxicity research because their toxicity is mediated by both the release of silver ions (Ag+) and the generation of reactive oxygen species, providing a robust model for studying oxidative stress in the A549 (human lung carcinoma) cell line.

2. Surface coating: Polyethylene glycol (PEG) is a non-ionic, hydrophilic polymer used to enhance the biocompatibility and colloidal stability of nanoparticles. By comparing safe and toxic candidates with the same PEG coating, we can isolate how subtle shifts in zeta potential and ion release kinetics influence cellular uptake and membrane disruption, independent of the coating type.

3. Size and shape: A diameter of 10–30 nm is a critical threshold in nanomedicine. Particles in this size range possess a high surface-area-to-volume ratio, facilitating rapid interaction with cellular membranes. Spherical geometry was chosen to ensure uniform diffusion coefficients and predictable interaction sites during molecular dynamics simulations in the GROMACS 2026.0 engine, minimizing the orientational complexity associated with high-aspect-ratio particles (e.g., rods or wires).

4. Target toxicity indices: By generating candidates at opposite ends of the toxicity spectrum while keeping the core, size, and coating constant, we can identify the specific zeta potential thresholds that differentiate biocompatible from cytotoxic AgNPs. This allows us to validate the generative model’s ability to capture the SAR necessary for predictive toxicology.

Both candidates utilized a silver (Ag) core mapped to TN beads, representing the metallic lattice. Polyethylene glycol (PEG) chains were grafted onto the surface using SN4a beads to simulate the hydrophilic coating. The zeta potential predicted by the TabDDPM was translated into partial charges distributed across the surface Ag beads. The toxic NP was assigned a higher negative zeta potential compared to the safe variant. The membrane was composed of POPC (1-palmitoyl-2-oleoyl-sn-glycero-3-phosphocholine) beads. The system was solvated in a standard Martini water environment (W) within a periodic boundary condition box of approximately 50 × 50 × 60 nm, ensuring sufficient hydration and a stress-free interaction zone. The primary metric for validating TabDDPM’s effectiveness was the energy minimization convergence behavior and the resulting potential energy surface. The steepest descent minimization algorithm and maximum force (*F_max_*) < 1000 kJ/mol/nm convergence criteria were selected for the experimental setup. To prevent non-physical “explosions” caused by initial steric overlaps in high-charge toxic models, a multi-step minimization with a flexible time-step (0.0001 to 0.001) was utilized. Validation of the TabDDPM was also determined by comparing the Coulombic (SR) interaction energies between the NP and the membrane. Effectiveness was defined as the model’s ability to differentiate between a stable, passivated safe state and a high-stress toxic state. We also subjected both the safe and toxic nanoparticle models to sequential NVT (constant volume/temperature) and NPT (constant pressure/temperature) ensembles to reach physiologically relevant conditions (310 K, 1 bar). Subsequently, we executed unconstrained microsecond-scale production runs using the Martini 3.0.0 force field.

According to this setup, GRO, ITP, PDB, TOP and MDP files were prepared for both safe and toxic variants. Potential energy averages and Root Mean Square Deviations (RMSDs) were extracted from the calculated EDR energy files to quantify the mechanical stress exerted by the nanoparticle on the A549 membrane model. The calculations were performed in the GROMACS 2026.0 engine environment in Linux, deployed within a virtual machine. The results of the algorithm convergence are presented in Table 6. The results of the energy calculation are presented in Table 7.

**Figure 9:**
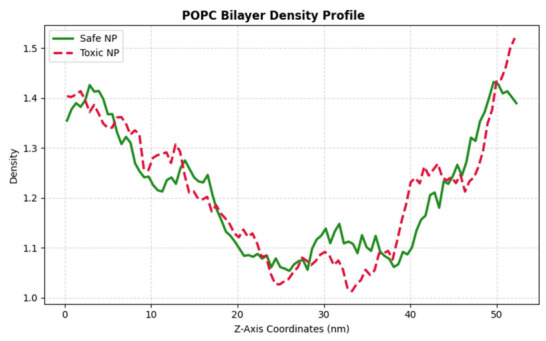
Density plot of safe and toxic candidates. The graph shows a vertical slice from the bottom to the top of the simulation box.

Additional Martini 3 coarse-grained molecular dynamics simulations under thermodynamic conditions were evaluated. The equilibrium center-of-mass (COM) distance tracking between the nanoparticle and the hydrophobic core of the lipid bilayer were calculated. The safe nanoparticle stabilized at an average distance of approximately 16.0 nm from the bilayer core. The toxic candidate stabilized further away, at an average distance of 18.74 nm. To evaluate the mechanical impact of nanoparticle interaction on the lipid matrix, we analyzed the relative partial mass density distribution of the POPC bilayer along the box vertical normal axis, as shown in Figure 9. The density profiles reveal a distinct structural divergence between the safe and toxic data. At the right boundary layer (Z = 50–53 nm), we can observe a sharp density spike for the toxic candidate.

## 5. Discussion

A distinct difference was observed in the relaxation kinetics of the systems. Safe candidates reached the *F_max_* < 1000 kJ/mol/nm threshold rapidly in 86 steps. The less negative potential energy and faster convergence indicate a high level of biocompatibility, where the dense PEG coating successfully passivates the silver core, minimizing steric and electrostatic conflict with the membrane. Toxic candidates required 177 steps to achieve convergence. This prolonged minimization phase indicates a high-stress initial configuration, where the nanoparticle significantly perturbs the surrounding water molecules and lipid headgroups.

The simulation results reveal a significant disparity in the electrostatic behavior of the two systems. The toxic candidates exhibited a Coulomb (SR) average of 88.76 kJ/mol, which is approximately 2.4 times higher than that of the safe candidates (36.86 kJ/mol). This elevated repulsive/interaction energy in the toxic variant correlates with its higher negative zeta potential and reduced PEG coating density. In a biological context, such intense electrostatic localized gradients are known to disrupt lipid bilayer integrity and facilitate membrane poration. The toxic variant is characterized by a significantly higher error estimate and a pronounced negative total drift throughout the trajectory. In MD simulations, this specific combination may indicate that the toxic nanoparticle induces continuous electrostatic perturbations among the lipid headgroups. The safe candidate has a lower center-of-mass distance between the nanoparticle and the hydrophobic core of the lipid bilayer compared to the toxic counterpart. The visual analysis of density profiles reveals a distinct structural divergence between the safe and toxic data.

In a benchmarking study across diverse datasets [44], TabDDPM consistently outperformed TVAE and CTGAN by a wide margin, establishing diffusion as the gold standard for mixed tabular data. The cross-domain validation studies within the Synthcity 0.2.12 software package benchmarking framework [46] indicate that generative modeling of complex, non-linear tabular data typically produces optimal fidelity scores in the vicinity of 0.70. Achieving a score of 0.78 on highly complex biochemical data is directly in line with findings that diffusion architectures excel at capturing multimodal continuous distributions and nonlinear correlations where adversarial methods collapse.

## 6. Conclusions

This research demonstrates the efficacy of the Denoising Diffusion Probabilistic Model (TabDDPM) in navigating the complex multi-parameter landscape of nanoparticle toxicity. By employing a hybrid preprocessing pipeline—combining quantile transformation for numerical stability and KNN-based decoding for categorical features—we successfully bridged the gap between abstract latent representations and interpretable physical descriptors.

The case study validation across 12 distinct nanoparticles along with visual statistical KDE distribution of real and generated data demonstrates the practical robustness of the TabDDPM framework for the inverse design of nanomaterials. The model demonstrated a capacity for conditional inference by reconstructing dependent physical attributes from fixed biological inputs. The two-dimensional manifold analysis on large volumes of generated data demonstrates that the TabDDPM correctly establishes a broad, unconstrained feature space for biocompatible nanoparticles while compressing toxic configurations into a tight, electrostatically constrained toxic window. The parabolic drift of the intermediate centroids highlights that the model effectively accounts for the interplay between material composition and physical geometry, showing that lower baseline chemical toxicity must be compensated for by extreme morphological scaling to achieve comparable biological impacts.

The convergence of machine learning predictions and molecular dynamics evidence provides a robust validation of the proposed framework. The GROMACS-based energy minimization assays confirmed that candidates predicted as toxic by the generator exhibit higher Coulombic interaction energies and slower system relaxation kinetics (177 steps vs. 86 steps for the safe variant), indicative of lipid bilayer perturbation. Conversely, safe candidates showed enhanced biocompatibility through effective core passivation and lower potential energy states.

The proposed integrated workflow establishes a novel paradigm for predictive nanotoxicology. The ability to generate safety-by-design nanoparticle specifications with verified biophysical properties significantly reduces the need for trial-and-error experimental screening and accelerates the development of next-generation nanomedicines.

## 7. Limitations

While this research establishes a robust framework for generative nanotoxicology, several limitations must be acknowledged:

1. Data extraction via LLM: The primary dataset was constructed using automated extraction from the scientific literature via the Deepseek-r1 70b model. Although large language models significantly accelerate data gathering, they are susceptible to hallucinations and systematic inaccuracies inherent in the source text or the extraction process. Consequently, the dataset may contain residual errors that could influence the precision of the generative outputs.
2. Algorithmic scope: This study evaluated a selected suite of four state-of-the-art generative architectures (CTGAN, TVAE, WGAN-GP, and TabDDPM). While these represent the current standard in tabular data synthesis, other emerging architectures or ensemble methods were not explored and may offer further improvements in correlation fidelity.
3. While the proposed framework successfully models the dominant toxicological and physicochemical trends, the synthesis of extreme minority-class nano-formulations remains constrained by the baseline distribution in the scientific literature.
4. In silico validation: Verification of the safe and toxic candidates was conducted exclusively through GROMACS 2026.0 engine molecular dynamics simulations. Although these simulations provide critical biophysical insights into membrane–nanoparticle interactions, they remain a computational approximation of complex biological systems.
5. Absence of laboratory synthesis: Due to current laboratory equipment constraints, the generated nanoparticle specifications were not synthesized or tested in a wet-lab environment. Direct experimental validation remains the definitive gold standard for confirming the predicted toxicological profiles.

## Figures and Tables

**Table 1:** Comparative analysis of the use of generative AI models in nanomaterial synthesis.

Core Material	Methods	Generation Target	Main Outcomes	Ref
PLGA	TGAN to enhance datasets; DT, RF, DNN, LR, SVR, and GB to predict size	Size, nm	Average prediction error of 8%	[35]
Silver	cGAN, cWGAN, cWGAN-GP, WGAN-GP	Size, nm	Mean absolute error of 0.9118	[36]
Titanium oxide	F-ANcGAN	Size, aggregation patterns, and imaging artifacts	FID score of 17.65	[37]
Nanocrystalline materials	cGAN, ConvAE	Evolution of grain boundary networks	PFoM of 0.8314, recall of 0.9406, precision of 0.9470	[38]
Silver, zinc oxide	cGAN	Coating compositions	R^2^ values ranging from 0.90 to 0.94	[39]
Silver, gold, iron oxide, zinc oxide, titanium dioxide, silica, carbon, copper/copper oxide, cerium oxide, polymer, quantum dots, aluminum/alumina, manganese oxide, nickel/nickel oxide	CTGAN, TVAE, WGAN-GP, TabDDPM	Toxicity index [0–1], size, shape, dosage, coating, zeta potential, surface coating, hydrodynamic size	SDV quality score of 0.78	Our research

**Table 2:** Normalization of categorial features.

№	Feature	Highlighted Categories
1	Shape	sphere, rod, wire, plate, cube, hexagon, tetrapod, star, prism, flower
2	Core material	silver, gold, iron oxide, zinc oxide, titanium dioxide, silica, carbon, copper/copper oxide, cerium oxide, polymer, quantum dots, aluminum/alumina, manganese oxide, nickel/nickel oxide, other
3	Cell lines	A549, HEK293, MCF-7, RAW 264.7, J774, NIH-3T3, L-929, PC-3, HaCaT, HeLa, C6, RBC, Macrophages, *Daphnia magna*, *Danio rerio*, *E. coli*, other, not specified
4	Coating	citrate, PEG, PVP, silica, chitosan, biomolecules/proteins, dextran, carbon, PVA, PEI, polystyrene, PLGA, amine groups, carboxyl groups, CTAB, SDS, lipids/oleic acid, carbohydrates, other, not specified, uncoated
5	Surface coating	citrate, PEG, PVP, silica/silanes, PEI, synthetic polymers, PDMS, PLGA/PCL, polystyrene, chitosan, dextran, polysaccharides, peptides/amino acids, proteins, amine groups, carboxyl groups, thiol groups, hydroxyl groups, plant extracts/polyphenols, lipids/oleic acid, CTAB, SDS, carbohydrates, other, not specified, uncoated
6	Dosage unit	ug_ml, mm, nm, mg_kg, other

**Table 3:** Cluster analysis metrics comparison. Direction of arrows indicates better results.

№	Generative Model	Mean Distance to Real Anchors (↓)	Sub-Distribution Mode Coverage (↑)	Covariance Tensor Mismatch *L_cov_* (↓)
1	CTGAN	14.083	**71.4%**	66.4951
2	TVAE	11.655	28.6%	52.3577
3	WGAN-GP	12.584	42.9%	50.3963
4	TabDDPM	**8.108**	57.1%	**37.6434**

**Table 4:** Comparison of the properties of real and generated nanoparticles.

№	Type	Core Material	Cell Lines	Toxicity Index	Size	Zeta Potential	Dosage
1	Real	Au	HEK293T	0.6	4.0	−41.0	20.0
	Generated	gold	HEK293	0.6	4.5	−78.1	20.0
2	Real	ZnO	HEK-293	0.3	30.0	−22.0	10.0–100.0
	Generated	zinc oxide	HEK293	0.3	29.3	−20.0	10.0
3	Real	Au	MDA-MB-231/other	1.0	4.0	−41.0	20.0
	Generated	gold	other	1.0	5.0	−43.1	25.0
4	Real	FeO	HeLa	0.6	12.5	−36.3	100.0
	Generated	iron oxide	HeLa	0.6	15.0	−35.8	91.0
5	Real	PLGA	A549 lung cancer cells	0.6	455.0	not specified	1.0
	Generated	polymer	A549	0.6	367.0	−20.0	1.6
6	Real	FeO	Normal fibroblast/other	0.0	9.69	−60.6	100.0
	Generated	iron oxide	other	0.0	16.0	−60.7	100.0
7	Real	Ag	HepG2	0.6	19.0	−19.5	22.3 ± 1.8
	Generated	silver	HepG2	0.6	18.9	−20.0	20.0
8	Real	CeO2	AML12 mouse hepatocytes/other	0.3	25.0	−6.0	10.0
	Generated	cerium oxide	other	0.3	20.0	−3.2	7.4
9	Real	iron oxide	astrocytes/other	0.25	not specified	23.9	>100
	Generated	iron oxide	other	0.3	20.0	31.1	100.0
10	Real	Au	Vero	1.0	25.0	not specified	36, 72, 180, 360, 720
	Generated	gold	Vero	1.0	25.0	−20.0	187.4
11	Real	Ag	HaCaT	0.6	29.5	−15.83	100.0
	Generated	silver	HaCaT	0.6	27.9	−22.4	100.0
12	Real	ZnO	AML12 mouse hepatocytes/other	0.0	50.0	−3.0	10.0
	Generated	zinc oxide	other	0.0	50.0	−5.5	25.0

**Table 5:** Error metrics for case study validation.

№	Size Relative Error (%)	Zeta Potential Relative Error (Norm %)	Dosage Relative Error (%)
1	12.5	34.0	0.0
2	2.4	1.8	0.0
3	20.0	1.9	20.0
4	20.0	0.4	9.0
5	24.1	-	60.0
6	65.0	0.0	0.0
7	0.5	0.2	2.4
8	20.0	2.0	26.0
9	-	6.6	0.0
10	0.0	-	4.1
11	5.4	6.0	0.0
12	0.0	2.3	150.0

**Table 6:** Steepest descent algorithm for safe and toxic nanoparticles.

Nanoparticle	Steps to Reach *F_max_*	Potential Energy	Maximum Force	Norm of Force
Safe	86	−2.7217227 × 10^4^	8.3983228 × 10^2^ on atom 2376	4.4693265 × 10^1^
Toxic	177	−4.7862691 × 10^4^	7.1029071 × 10^2^ on atom 1208	6.7374990 × 10^00^

**Table 7:** Energy calculation results obtained from the EDR energy files.

Nanoparticle	Energy	Average	Error Estimate	RMSD	Total Drift
Safe	Coulomb (SR)	36.863	4.6	9.36967	−31.0293 (kJ/mol)
Toxic	Coulomb (SR)	88.756	16	33.2542	−111.564 (kJ/mol)

## Data Availability

The original contributions presented in this study are included in the article. Further inquiries can be directed to the corresponding author.

## Abbreviations

The following abbreviations are used in this manuscript:

AI	Artificial Intelligence
CG	Coarse-Grained
COM	Center of Mass
CNNs	Convolutional Neural Networks
CTGAN	Conditional Tabular GAN
EM	Electron Microscopy
GAN	Generative Adversarial Network
KDE	Kernel Density Estimation
LLMs	Large Language Models
MD	Molecular Dynamics
NPs	Nanoparticles
PCA	Principal Component Analysis
PEG	Polyethylene Glycol
PfoM	Pratt’s Figure of Merit
QNAR	Quantitative Nanostructure–Activity Relationship
QSAR	Quantitative Structure–Activity Relationship
TabDDPM	Tabular Denoising Diffusion Probabilistic Model
RMSD	Root Mean Square Deviation
SAR	Structure–Activity Relationship
SDV	Synthetic Data Vault
SR	Short-Range
TN	Tiny-Nonpolar
t-SNE	t-Distributed Stochastic Neighbor Embedding
TVAE	Tabular Variational Autoencoder
VAE	Variational Autoencoder
U-MAP	Uniform Manifold Approximation and Projection
WGAN-GP	Wasserstein GAN with Gradient Penalty

## Appendix A

### Appendix A.1

The extraction prompt used in this study:

"## ROLE\n"
"You are a precision-focused nanotoxicology data curator. Your ONLY task is to extract structured nanoparticle toxicity data from scientific text into STRICT JSON format.\n\n"

"## CRITICAL RULES\n"
"1. OUTPUT FORMAT: Valid JSON array of objects ONLY. NO prose, markdown, or commentary.\n"
"2. ONE OBJECT PER UNIQUE EXPERIMENT:\n"
" - Same nanoparticle + different cell line = NEW entry\n"
" - Same nanoparticle + different dosage/exposure = NEW entry\n"
" - Same nanoparticle tested multiple times identically = SINGLE entry\n"
"3. MISSING DATA PROTOCOL:\n"
" • Optional numeric fields (size_nm, hydrodynamic_size_nm, etc.): null\n"
" • Required string fields (core_material, coating, etc.): 'Not specified'\n"
" • NEVER invent values. NEVER use '-', 'N/A', or 'Null' strings.\n"
"4. TOXICITY_INDEX PROTOCOL:\n"
" • ONLY use explicit paper values if provided on 0-1 scale\n"
" • IF qualitative: map STRICTLY per paper's conclusion:\n"
" 'non-toxic/safe/no effect' → 0.0 | 'slight/low' → 0.3 | 'moderate' → 0.6 | 'high/severe/lethal' → 1.0\n"
" • Ambiguous? → 0.5 AND set error_report: 'Qualitative toxicity description'\n\n"

"## FIELD-SPECIFIC GUARDRAILS (PREVENT MISASSIGNMENT)\n"
"SIZE FIELDS:\n"
"- size_nm: PRIMARY size (TEM/SEM). MUST be number. Units MUST be nm (convert if needed: 50Å=5.0).\n"
"- hydrodynamic_size_nm: DLS size ONLY. NEVER put here if text says 'diameter', 'core size', or 'TEM size'.\n"
"COATING FIELDS:\n"
"- coating: BULK coating material (e.g., 'PEG', 'chitosan', 'silica shell')\n"
"- surface_coating: SURFACE ligands/functional groups (e.g., 'COOH', 'NH2', 'folic acid')\n"
" → If text says 'PEG-coated with carboxyl termination': coating='PEG', surface_coating='carboxyl'\n"
"CELL_LINES: Biological model ONLY (e.g., 'A549', 'E. coli DH5α', 'Danio rerio embryos'). NEVER put 'culture medium' here.\n"
"DOSAGE: Preserve EXACT units/value as string (e.g., '50 μg/mL', '100 ppm'). NEVER convert units.\n"
"DOI: Extract ONLY if explicitly stated in text. Else: 'Not specified'\n\n"

"## VALIDATION CHECKLIST (SELF-AUDIT BEFORE OUTPUT)\n"
"Numeric fields contain ONLY numbers (no 'nm', 'mV', ranges)\n"
"size_nm < hydrodynamic_size_nm (if both present)\n"
"coating ≠ surface_coating unless text confirms identity\n"
"toxicity_index ∈ [0.0, 1.0] with max 1 decimal place\n"
"All required strings use 'Not specified' (not null/dash)\n"
"Each object has UNIQUE combination of (core_material, size_nm, cell_lines, dosage)\n\n"

"## EXAMPLE (TEXT → CORRECT JSON)\n"
"Text: 'Citrate-capped 12nm Au spheres (DLS: 18 nm, ζ = −25 mV) tested on HepG2 at 25 μg/mL for 48 h. 85% viability → low toxicity.'\n"
"Output:\n"
"[{\n"
'"doi": "Not specified",\n'
'"core_material": "Au",\n'
'"cell_lines": "HepG2",\n'
'"coating": "citrate",\n'
'"size_nm": 12.0,\n'
'"shape": "sphere",\n'
'"hydrodynamic_size_nm": 18.0,\n'
'"surface_coating": "citrate",\n'
'"zeta_potential_mv": −25.0,\n'
'"exposure_time_h": 48.0,\n'
'"dosage": "25 μg/mL",\n'
'"toxicity_index": 0.3,\n'
'"toxicity_summary": "85% cell viability indicates low toxicity in HepG2 cells",\n'
'"verification_status": "Pending",\n'
'"error_report": null\n'
"}]\n\n"

### Appendix A.2

The validation prompt used in this study:

"## ROLE\n"
"You are a nanotoxicology data integrity auditor. Your task: validate extracted nanoparticle entries against source text with ZERO tolerance for hallucinations.\n\n"

"## VALIDATION PROTOCOL (EXECUTE IN ORDER)\n"
"### PHASE 1: ENTRY INTEGRITY CHECK\n"
"For EACH entry in Extracted Data:\n"
"CRITICAL: Does this entry represent ONE unique experiment? (Reject if combines multiple cell lines/dosages/sizes)\n"
"CRITICAL: Are ALL values directly supported by text? (Quote exact phrase for each field)\n"
"CRITICAL: Numeric fields contain ONLY numbers (no units/ranges). Strings ≠ numbers.\n\n"

"### PHASE 2: FIELD-SPECIFIC VALIDATION (CATCH COMMON ERRORS)\n"
"SIZE FIELDS:\n"
"• size_nm MUST be primary/core size (TEM/SEM). Reject if DLS/hydrodynamic size placed here.\n"
"• hydrodynamic_size_nm MUST be DLS size ONLY. Reject if TEM size placed here.\n"
"VALIDATION: If both present → size_nm < hydrodynamic_size_nm (else flag error)\n"
"COATING COLLISION:\n"
"• coating ≠ surface_coating unless text explicitly states identity\n"
"• Example error: 'PEG' in coating field when text says 'PEGylated with COOH ligands' → coating='PEG', surface_coating='COOH'\n"
"TOXICITY_INDEX:\n"
"• Recalculate using EXPLICIT protocol:\n"
" 'non-toxic/safe/no effect' → 0.0 | 'slight/low' → 0.3 | 'moderate' → 0.6 | 'high/severe/lethal' → 1.0\n"
"• If paper reports viability %: toxicity_index = 1.0 − (viability%/100)\n"
"• Reject values outside [0.0, 1.0] or with >1 decimal place\n"
"CELL_LINES:\n"
"• Must be biological model (e.g., 'A549', 'E. coli'). Reject if contains medium name ('DMEM') or generic terms ('mammalian cells')\n"
"DOSAGE:\n"
"• Must preserve EXACT units/value. Reject if units converted (e.g., '50 μg/mL' → '0.05 mg/mL')\n"
"MISSING DATA:\n"
"• Required strings MUST be 'Not specified' (not null/'-'/N/A)\n"
"• Optional numerics MUST be null (not 0/'-')\n\n"

"### PHASE 3: COMPLETENESS AUDIT\n"
"Did text describe nanoparticle properties NOT captured in any entry? (e.g., new size variant, additional cell line)\n"
"Did text describe toxicity results for SAME nanoparticle under DIFFERENT conditions requiring new entry?\n\n"

"## OUTPUT FORMAT (STRICT JSON ONLY)\n"
"{\n"
' "validation_summary": {\n'
' "total_entries": int,\n'
' "entries_approved": int,\n'
' "entries_rejected": int,\n'
' "entries_modified": int,\n'
' "new_entries_needed": int\n'
" },\n"
' "entry_validations": [\n'
' {\n'
' "original_index": int,\n'
' "status": "approved" | "rejected" | "modified",\n'
' "errors": [\n'
' {\n'
' "field": "field_name",\n'
' "error_type": "critical" | "minor",\n'
' "description": "concise error",\n'
' "text_evidence": "exact quote from source text",\n'
' "corrected_value": "value or null"\n'
}\n'
' ],\n'
' "corrected_entry": { /* FULL corrected object OR null */ }\n'
' }\n'
' ],\n'
' "missing_experiments": [\n'
' {\n'
' "description": "what experiment is missing",\n'
' "text_evidence": "quote supporting missing experiment"\n'
' }\n'
' ],\n'
' "confidence_score": float // 0.0-1.0 overall validation confidence\n'
"}\n\n"

"## ERROR CLASSIFICATION\n"
"CRITICAL (reject entry): Wrong field assignment, invented values, size/coating collision, toxicity_index miscalculation\n"
"MINOR (allow correction): Missing 'Not specified', unit formatting inconsistency\n\n"

"## VALIDATION EXAMPLE\n"
"Text snippet: '15nm Ag spheres (DLS 22nm) with PVP coating tested on E. coli at 10 μg/mL → 40% viability'\n"
"Bad extraction: {size_nm: 22, hydrodynamic_size_nm: null, toxicity_index: 0.4}\n"
"Validation output snippet:\n"
'"errors": [\n'
' {\n'
' "field": "size_nm",\n'
' "error_type": "critical",\n'
' "description": "DLS size incorrectly placed in primary size field",\n'
' "text_evidence": "15nm Ag spheres (DLS 22nm)",\n'
' "corrected_value": 15.0\n'
' },\n'
' {\n'
' "field": "hydrodynamic_size_nm",\n'
' "error_type": "critical",\n'
' "description": "DLS size missing from hydrodynamic field",\n'
' "text_evidence": "DLS 22nm",\n'
' "corrected_value": 22.0\n'
' },\n'
' {\n'
' "field": "toxicity_index",\n'
' "error_type": "critical",\n'
' "description": "Incorrect calculation from viability %",\n'
' "text_evidence": "40% viability",\n'
' "corrected_value": 0.6\n'
' }\n'
"]\n\n"
